# The Costs of Healthcare in Prison and Custody: Systematic Review of Current Estimates and Proposed Guidelines for Future Reporting

**DOI:** 10.3389/fpsyt.2018.00716

**Published:** 2018-12-20

**Authors:** Shivpriya Sridhar, Robert Cornish, Seena Fazel

**Affiliations:** ^1^College of Arts and Sciences, University of North Carolina at Chapel Hill, Chapel Hill, NC, United States; ^2^Oxford Health NHS Foundation Trust, Oxford, United Kingdom; ^3^Department of Forensic Psychiatry, University of Oxford, Oxford, United Kingdom

**Keywords:** prison, custody, detention, costs, expenditure, guidelines, healthcare services

## Abstract

**Aims:** We aimed to review prison healthcare expenditure internationally.

**Objectives:** To systematically review healthcare spending on prisoners worldwide, examine comparability between countries, and develop guidelines to improve reporting.

**Methods:** Five bibliographic indexes (International Monetary Fund, ProQuest: Statistical Abstracts of the World, PubMed, Google Scholar, and JSTOR) were searched for the costs of prison and prison healthcare, supplemented with country-specific searches for the 20 countries with the highest prison populations. Information on overall healthcare costs, their breakdown by categories, and their proportion to overall prison expenditure was extracted. PRISMA guidelines were followed.

**Results:** Prison healthcare expenditure data was identified for 10 countries, and overall operating costs were reported for 12 countries. The most commonly reported healthcare cost was for primary medical care. Healthcare costs reporting varied widely, and few countries were comparable. We developed a set of guidelines for consistent and transparent reporting of healthcare costs.

**Conclusions:** Few countries report the costs of healthcare services in prison. When reported, there is a lack of clarity and consistency as to what is included. Using the proposed reporting guidelines would enable national trends and international comparisons to be investigated, and any recommended benchmarks to be monitored.

## Introduction

Healthcare in prison varies widely across countries, and differences in service provision contribute to morbidity and mortality outcomes inside custody (1) and on release (2). Many studies have examined disease prevalence rates, and prevention, care, and treatment in prison (3, 4), but the current evidence base lacks information on the costs of healthcare services. Combined with information on the prevalence of healthcare problems, international comparisons of prison health expenditure could better inform decisions on the levels of appropriate funding, enable benchmarks to be monitored, and allow for planning of service provision.

Information on the costs of operating prisons have been published since the 1950s (5), including a 2004 report of international costs (6). However, no review of prison healthcare costs across countries currently exists. Government and other reports of healthcare costs in prisons have been published, but it is not clear in reporting what particular services are included, which examine variously “medical care” (7), “prison hospitals” (8), or “prisoner health” (9). Furthermore, what is included within these categories is not clear. Scotland, for instance, lists mental health and dental care as prison healthcare expenditures (10), while Australia provides only a single overarching figure for prisoner health (9).

In this systematic review, we have aimed to provide an international overview of the annual costs of prisoner healthcare, what healthcare services are included in the identified reports, and calculate the proportion of overall prison operating budgets allocated to health. In addition, we develop and propose reporting guidelines.

## Methods

### Search Strategy

We searched IMF, ProQuest: Statistical Abstracts of the World, Google Scholar, JSTOR, and PubMed databases. We performed non-country specific searches, with a combination of keywords: “prison” OR “justice,” AND “expenditure,” “spending,” OR “costs” (Table 1 for details). Then, additional targeted searches on Google Web and government databases (e.g., Statistics Canada) were conducted for the 20 countries with the largest prison population (11) and countries included in a previous review of recidivism rates (12), with a combination of keywords: country name AND “prison healthcare cost,” country name AND “ministry of justice,” country name AND “prison” AND “expenditure” OR “service.” No language or publication date restrictions were set, and the most recent and relevant cost report was identified. When necessary, correctional services were contacted to clarify data. A review protocol [CRD42018102534] was submitted and published on the PROSPERO register of systematic review during data extraction.

**Table 1:** Database Search Strategies.

**Database**	**Search terms**	**Additional criteria**
IMF datasets	“prison” AND “expenditure” OR “prison” AND “costs”	Time series datasets (no Filters used) (classified as “Budgetary Central Government” OR “Extrabudgetary Central Government” OR “General Government” OR “Local Governments” OR “Social Security Funds” OR “State Governments” OR “Central Government” + in “Domestic Currency” OR “Percent of GDP” + “Expenditure on Prisons”
ProQuest: Statistical Abstracts of the World	“prison” AND “expenditure” OR “justice” AND “expenditure” OR “prison” AND “health”	No Applied Filters
JSTOR	“prison spending” OR “prison expenditure” OR “prison costs”	No restrictions
Google Scholar	“prison expenditure” OR “prison service spending”	No restrictions
PubMed	(“prisons”[MeSH Terms] OR “prisons”[All Fields] OR “prison”[All Fields]) AND (“health expenditures”[MeSH Terms] OR (“health”[All Fields] AND “expenditures”[All Fields]) OR “health expenditures”[All Fields] OR “expenditure”[All Fields])	No restrictions

Per-prisoner estimates for each country were calculated as follows:

1. Identifying total expenditures from the included reports (excluding the US and Germany, which reported per-prisoner expenditure based on all state prison populations)
2. Calculation of per-prisoner cost (using the prison population from World Prison Brief statistics) (13). If the prison population was not available for the same year as the cost report, the following year's prison population was used;
3. Conversion of per-prisoner cost estimate into inflation-adjusted 2016 International US dollars (using “CCEMG-EPPI-Center Cost Converter” external database). Stage 1 and 2 computational values (GDP deflator and PPP) of the validated conversion tool were obtained from IMF World Economic Outlook Database. Costs were first adjusted for inflation within original economy [using GDP deflator values], and then to purchasing power parity/price level between countries (14).

The cost conversions in this review may be limited by using purchasing power parities for Gross Domestic Product, which cover a broader array of goods and services than context-specific purchasing power parities (e.g., healthcare or technology purchasing power parities). However, based on the heterogeneity of costs and inventories reported by countries, purchasing power parities for Gross Domestic Product appeared to be more fitting for this review than context-specific conversion factors (14).

### Study Design

#### Geographical

Official national and regional data were extracted from search engines. Unofficial regional data was found for one country (15), but was not included.

#### Data Items

The measurements and descriptions of economic costs were limited to direct prison operating costs and/or healthcare costs. Studies reporting indirect costs to prisoners (e.g., productivity loss) were excluded. The most recent information was used. Most countries presented financial accounts for the 2015–16 or 2016–17 periods, so all cost outcomes were indexed to 2016 International US dollars.

#### Data Sources

Financial reports on prisons/correctional services solely were included. Datasets which did not specify relevant outcome measurements (i.e., prison operations, healthcare) or the population (i.e., whole country, adult prisoners) were excluded. Expenditure reporting on police, courts, or other services affiliated with Ministries of Justice was excluded, as were assessments of prisoner health services separate from expenditure data (16). Governments typically separate prison expenditures into capital and operating costs. Capital costs refer to fixed, one-time investments on buildings, equipment, or land. Operating costs of prisons refer to day-to-day running costs, accommodation-related costs, and staff costs (17). Ideally, prison healthcare broadly refers to the services in place to address health needs and reduce health risks. It can include primary and psychiatric care, and specialized services. Information for two other countries (Greece and Switzerland) were available but not used. They were not from official governmental sources, did not specify constituent elements of costs, and are likely to be underestimates (7, 15). Costs for Sweden did not include infrastructure costs and were also not included (18).

#### Data Extraction

One of the authors (SS) screened and extracted the data, excluding publications and data points that did not report costs, and determined the remit of the data (e.g., national, regional).

#### Data Analysis

Meta-analysis was not conducted due to heterogeneity in definition and measurement of cost outcomes.

### Subgroup Comparison

**Figure 1:**
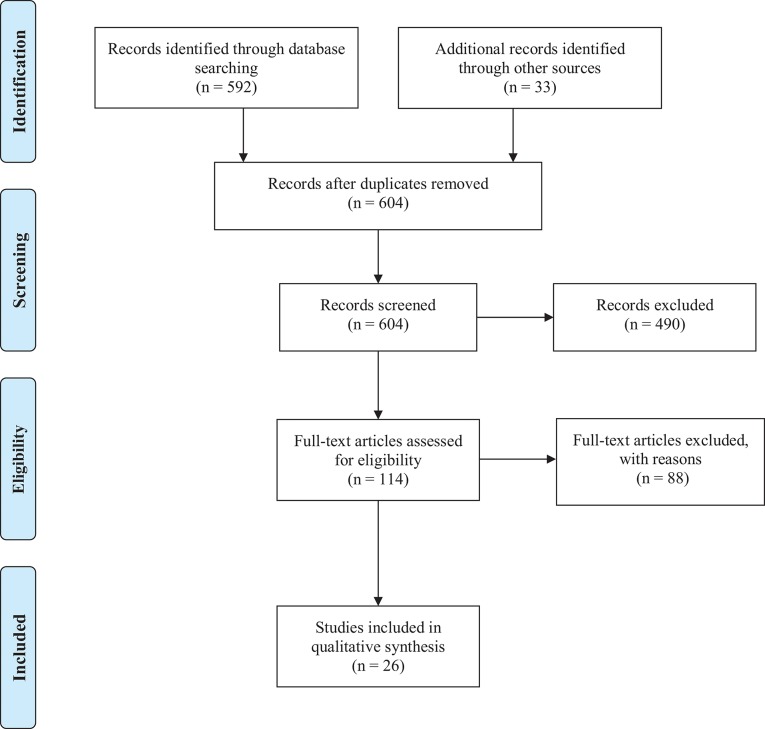
PRISMA flowchart.

We compared healthcare cost descriptions by country to account for differences in categorization of expenses. Descriptions for the most recent period are included. PRISMA guidelines were followed (Table S1, Figure 1). SS and SF assessed primary outcome measures of healthcare service costs and summary descriptions.

## Results

We identified official country reports of prison healthcare costs for 10 countries (Table 2).

**Table 2:** Country reports.

**Country**	**Publisher**	**Source**	**Year of reporting**	**Expenditure category**	**Sample**
Sri Lanka	Ministry of Health, Nutrition and Indigenous Medicine	National Health Accounts	2013	Health	National prison hospitals (central government-financed)
Romania	Ministry of Justice	Ministry of Justice	2016	Health	National Penitentiary System
India	Ministry of Home Affairs	National Crime Records Bureau State/UT Prison Headquarters	2015	Health and Operating	State Prisons/Jails (“prisons” and “jails” used interchangeably)
South Africa	Department of Correctional Services (DCS)	Annual Report of Department of Correctional Services	2016–17	Health and Operating	DCS National Facilities
Ireland	Irish Prison Service	Irish Prison Service	2013	Health	National - All prisons (open, closed, and high security), including pre-trial detainees
	Irish Prison Service	Irish Prison Service	2015	Operating	National - All prisons (open, closed, and high security), including pre-trial detainees
Belgium	Belgian Health Care Knowledge Centre (KCE)	KCE Report 293Cs	2015	Health	National - All Belgian prisons (35 prisons recorded in 2015)
Australia	Productivity Commission	Productivity Commission for the Steering Committee for the Review of Government Service Provision	2015–16	Health and Operating	National - Department of Correctional Services
UK: Scotland	The Scottish Parliament	Health and Sport Committee	2016–17	Health	National - Scottish Prison Service (15 prisons)
	Scottish Prison Service	Audit Scotland	2015–16	Operating	National - Scottish Prison Service (15 prisons)
United States	PEW Charitable Trusts	PEW, Vera Institute of Justice, and state officials	2015	Health	State prisons (and jail populations of five states)
	Bureau of Justice Statistics	Department of Justice	2001	Operating	State Prisons
UK: England and Wales	National Audit Office	Comptroller and Auditor General	2016–17	Health	National
	Ministry of Justice	Ministry of Justice and HM Prison and Probation Services	2016–17	Operating/Direct	National
France	Ministry of Justice	Directorate of Prison Administration	2015	Operating	National
Singapore	Ministry of Home Affairs	Ministry of Home Affairs	2016 FY	Operating	National
Canada	Statistics Canada	Statistics Canada	2016–17	Operating	Provinces and Territories
Germany	The Berlin Senate Department for Justice and Consumer Protection	The Berlin Senate Department for Justice and Consumer Protection	2013	Operating	All German States
New Zealand	Department of Corrections	Department of Corrections	2015–16	Operating	National
UK: Northern Ireland	Northern Ireland Prison Service (NIPS)	NIPS	2016–17	Operating	National

Annual healthcare expenditures per prisoner ranged from approximately $34 per year to $6,714 per year (Table 3). There was also wide variation in the percentage of operating costs attributable to healthcare, ranging from 2 to 18% (Table 4).

**Table 3:** Reported annual healthcare expenditure per prisoner.

**Country**	**Per prisoner cost (2016 US$)**	**Year of reporting**
Sri Lanka (8)	$34	2013
Romania (19)	$103	2016
India (20)	$109	2015–16
South Africa (21)	$1,001	2016–17
Ireland (22)	$2,932	2013
Belgium (23)	$4,748	2015
Australia (9]	$5,096	2015–16
UK: Scotland (10)	$5,288	2016–17
United States (24)	$5,720	2015
UK: England and Wales (25)	$6,714	2016–17

**Table 4:** Annual overall prison operating costs and healthcare expenditure as percentage of operating expenditures.

**Country**	**Annual operating expenditure per prisoner (indexed to 2016)**	**Healthcare expenditure as % of operating expenditure**
India (20)	$5,900	2%
South Africa (21)	$22,412	4%
United States [State Prisons] (24, 26)	$29,978	18%
France (27)	$44,410	N/A
Singapore (28)	$48,406	N/A
Canada [Provincial, State, Territorial] (29)	$49,251	N/A
UK: England and Wales (25, 30)	$50,675	13%
Australia (9)	$56,786	9%
Germany (31)	$57,380	N/A
UK: Scotland (10, 32)	$60,943	9%
New Zealand (17)	$65,336	N/A
Ireland (22, 33)	$68,019	4%
Northern Ireland (34)	$76,516	N/A

Seven countries described the constituent elements of healthcare spending, but these were not comparable (Table 5). The most commonly reported cost outcome was “primary medical care,” followed together by “medical supplies” (mostly medication) and “mental health services.” In the three countries (Sri Lanka, Ireland, Australia) that did not clarify expenditures, categorization varied from “curative care” (8) to “medical care” (22) to “prisoner health costs” (9). Most countries did not clarify how expenditure was allocated across services.

**Table 5:** Healthcare expenditure descriptions.

	**Primary medical Care**	**Medical supplies (incl. medicines)**	**Mental Health services (incl. visiting psychologists/psychiatrist)**	**Staff (incl. doctors, nurses, pharmacists)**	**Diagnostic Tests (incl. lab services and screening)**	**Dental**	**Substance abuse treatment**	**Consultants (incl. External psychiatric, gynecological, dental, optical)**	**Nursing care**	**Hygiene**	Other^*^
Sri Lanka (8)											X
Romania (19)		X								X	X
India (28)	X		X	X	X						X
South Africa (21)	X	X			X					X	X
Ireland (22)											
Belgium (23)	X	X	X	X		X		X	X		X
UK: Scotland (10)	X	X	X	X	X	X	X	X	X		
Australia (9)											X
United States (24)	X	X	X	X		X	X				X
UK: England & Wales (25)	X		X				X				

* *see Table S2 for full inclusion and exclusion details*. In India, health services include “visiting psychologists/psychiatrists.”

## Discussion

In this systematic review, we identified 10 countries that have reported healthcare costs in prisons. In addition, we were able to calculate the proportion of overall operating expenditure allocated to healthcare in 7 countries. Among the 20 countries with the highest prison populations (11), only 4 reported operating and healthcare expenditures on prisoners (20, 21, 24, 25). There were large variations in how healthcare costs were defined across countries, and consistency and transparency is required to enable international comparison. We have sought to address this by developing guidelines for future reporting.

One other finding was that only two countries reported spending more than 10% of their overall prison operating budgets on prison health (24, 25). Additionally, we did not find any clear links between overall spending and the proportion on healthcare. Notably, Ireland had the highest overall operating expenditure per prisoner among the countries reporting healthcare costs, but only 4% of its budget was allocated toward healthcare, which put it among the countries with the lowest proportion (22, 33).

Seven of the 10 countries that reported on healthcare expenditure provided specific detail on the services provided. The breakdown of these services varied between countries. It was not clear in some cases whether staff expenses included salaries of allied health professionals such as pharmacists, technicians, dentists, and visiting doctors (23), or whether “medical supplies” included medicines and medical equipment (21). Dental services lacked sufficient detail (10, 24), and mental health services were defined inconsistently across countries, with some countries integrating addiction care into mental health services (10, 24, 25), but another not (20).

One main implication of these findings is that international comparisons of healthcare expenditures on prisoners are currently not possible due to lack of consistency and transparency in reporting. To address this, we have developed a brief checklist covering definitions, inclusion, and exclusion criteria, and sources of funding that contribute to prison healthcare (such as national health services or ministry of justice) (Table 6). This checklist follows the structure of existing national financial audits, and is based on examples of good practice based on the current review, those categories that appear to be most consistently reported, and previous context-specific reporting guidelines (12, 15, 24, 36). The current proposed checklist would enable international comparison of overall healthcare expenditure in prisons, and provide country specific breakdowns as to how resources are allocated. Consistent international reporting would assist in monitoring whether countries meet basic standards for prisoner health, and allow for examination of links between adverse health outcomes in prisoners and variation in services provided. It would also help to develop and monitor recommended levels of appropriate funding. Different governments are likely to have competing additional priorities of healthcare need (20, 21), which will be reflected in how resources are spent and be reported in this checklist. Systematic reporting of expenditure may lead to improved health outcomes for prisoners by, for example, targeting specific areas of need in future service provision. They could also lead to increased efficiency and a greater focus on prevention. We recommend that prison services include the checklist information in annual accounts, where expenditures are often separated by service category and funding source (37). We have sought to streamline these guidelines to assist in feasibility of completion, standardization, and integration into national financial audits.

**Table 6:** Prison healthcare expenditure reporting checklist.

**COUNTRY**		
Time Period:	-For which financial year(s) are you reporting?	
Prisons Operating Expenditure:	-Does this include depreciation, financial payments on capital assets, fixed assets, and/or transfers/subsidies? *(Recommendation: exclude these costs)*	
Prisons Health Expenditure:	-What was the annual expenditure on physical and mental healthcare?	
% Operating Budget allocated to Medical Care:	-What percentage of the overall budget allocated to prisons went toward prisoner medical/healthcare?	
**PRISONER POPULATION**		
**I. Context/Demographics:**		**Guidelines**
a. Geographical	Are you reporting prison expenditures for the whole country, or a particular region?	Report whole country
b. Sample size	Which prison population(s) are being sampled and accounted for in expenditure reporting?	Include *all* adult (18 and older) private, public, and remand /detainees/unconvicted prisoners Include federal and provincial/regional prison institutions
c. Characteristics	Age, gender, ethnicity, health insurance status	Report age distribution in 10-year increments, natal gender, white vs. non-white ethnicity and health insurance status of incarcerated persons
d. Length of current sentence	Distribution of offense types and sentence length (35)	Categories: violence (including robbery), sex offense (contact vs. non-contact), burglary/theft, drug offense, other Report sentence length in following bands: < 1 year, 1–3 years, 4–9 years, >10 years, life sentence
**II. Exclusion Criteria**	
a. Other samples	Which samples are not included in financial accounting?	Exclude prisoners under 18 years of age, and those in immigration detention centers and police custody, and persons in *external* secure mental health facilities
**COST OUTCOMES**		
**I. Priority Areas**		**Guidelines**
a. Medical Supplies	How much money was allocated to medical supplies for prisoners? List goods included.	Include expenditures on pharmaceuticals/drugs, disability aids, vaccines, and equipment *maintenance* (separately if possible)
b. Healthcare Personnel	How much was spent on payroll services?	Report total amount spent on salaries of doctors, nurses, pharmacists, and technicians
	How much was spent on non-payroll services?	Report expenditures on visiting psychiatrists and external consultants
c. Diagnostic: Screenings and Tests	How much was spent on screenings for diseases in prisons? What types of diagnostic tests are offered?	Report expenditures on TB/AIDS/other screenings and laboratory/imaging tests separately
d. Primary Care	How much was spent on primary care services for prisons?	Report expenditures on GP Medical care and Nursing care separately
e. Psychiatric	How much money was allocated to psychiatric services in prisons?	Report expenditure on mental health programs and services
f. Rehabilitation	Is rehabilitative *healthcare* included in prison expenditure? If yes, how much was spent?	Report expenditure on substance abuse treatment *only*
g. Dental	Are dental services included in prison expenditure? If yes, how much was spent?	Report expenditure on dental services (e.g., general cleaning, orthodontics)
h. Optical	Are optical services included in prison expenditure? If yes, how much was spent?	Report expenditure on optical services (e.g., eye exams, glasses)
i. Maternal and Child	Are maternal and child health services included in prison expenditure? If yes, what services are provided and how much was spent?	Report expenditure on maternal and child health services (e.g., nutrition)
j. Surgical Procedures	Are any surgical procedures included in prison expenditure? If yes, how much was spent?	Report expenditures on any surgical procedures
k. Specialized Services	Are any other specialized *on-site* services included in prison expenditure? If yes, how much was spent?	Report expenditures on Gynecological, Palliative, Physiotherapy, Chemotherapy/Radiation, Occupational health services (if offered in prison)
l. Other	Are there any other health-related expenses?	Example: Hospitalizations, Hygiene
**II. Exclusion Criteria**		**Guidelines**
a. Capital Costs	Medical Equipment, Depreciation, Payments on assets	Example: How much was spent on procedural equipment, lab/imaging equipment, or payments on assets?
b. Social Services	Spiritual and social work services	Example: Religious ministry
c. Transport	Transport costs	Exclude ambulatory stay costs (i.e. emergency hospital stays following use of an ambulance)
d. Off-Site	Specialized outpatient care	Exclude care unavailable on-site
e. Other	Are there any other costs excluded from calculation?	
**FUNDING SOURCES**		
**I. Funding Agencies**		
a. National Prison Service/Department of Justice	What amount or percent of healthcare expenditures in prisons is allocated by prison services/justice departments?	
b. National Health Services	What amount or percent of healthcare expenditures in prisons is allocated by national health services?	
c. Other	Are there any other systems of financing prison healthcare in your country (e.g., private insurance)?	

## Limitations

Using percentage allocations of overall operating expenditure allocated to healthcare does not necessarily allow for cross-country comparison. For example, Australia and Scotland made similar allocations, of ~9% (9, 10). However, due to the different descriptions of services provided, it is unclear whether the two countries provide equivalent healthcare services for prisoners. Some country estimates were based on limited data. For example, calculations for the US and Canada were limited by exclusion of federal prisons, constituting 19.9% of Canadian adult institutions and 7.9% of combined American state and federal institutions (13). There were variations in American prisons with regard to sources of funding, which may have underestimated costs in some states. For example, inpatient hospitalization costs were being met by insurance companies in some cases, but borne by the prison directly in others. Similarly, in some states, community mental health services covered some of the cost of prisoner treatment (24).

There are additional sources of information on healthcare services provided in prisons which we have not included in this review, as they do not provide detail on expenditure (16). Future work may look at such reviews of prison healthcare (for non-financial purposes) to provide a more detailed assessment of what services are provided, in conjunction with examining financial reporting.

## Author Contributions

SF and SS conceived and designed the experiments. SF and SS performed the experiments. SS analyzed the data. SF, SS, and RC wrote the paper. SF supervised the project.

### Conflict of Interest Statement

The authors declare that the research was conducted in the absence of any commercial or financial relationships that could be construed as a potential conflict of interest.
