# Supplementary material for: The Costs of Healthcare in Prison and Custody: Systematic Review of Current Estimates and Proposed Guidelines for Future Reporting
Source: Front Psychiatry. 2018 Dec 20;9:716. doi: 10.3389/fpsyt.2018.00716 (PMC6306428; doi:10.3389/fpsyt.2018.00716)
Supplement: Supplementary file 2 [file Table_2.docx]

S2 Table. Healthcare Expenditure Descriptions

| Country | Included | Excluded | Uncertainties |
| --- | --- | --- | --- |
| Sri Lanka | Prison Hospitals: Specialized in-patient, Rehabilitative care | -Laboratory/Imaging services  -Optical and hearing aids  -Other | No qualification of rehabilitative care |
| Romania | -Medical Supplies  -Hygiene  Other: Disinfectants |  | Breakdown of services |
| India | -Staff  -Primary medical care  -Visiting psychologists  -Diagnostic tests  Other: In-patient treatment |  | Not representative of prisons in *all* states |
| South Africa | -Medical supplies (meds, vaccines)  -Primary medical care  -Diagnostic tests  -Hygiene  Other: Nutrition |  | Psychological care integrated into spiritual, reintegration and social work services, rather than healthcare services |
| Ireland | Medical |  | Breakdown of services |
| Belgium | -Primary medical care  -Mental health care  -Medical Supplies/Pharmacy  -Staff  -Consultants [External]  -Nursing Care  -Dental Care  Other: Secondary Care |  | ‘Delivery and treatment’ and ‘health care organization’ constituent costs uncertain |
| UK: Scotland | -Medical supplies  -Staff  -Primary medical care  -Mental Health  -Diagnostic tests  -Consultants [Psychiatric, Dental, Optical]  -Nursing care [Primary]  -Substance abuse treatment |  |  |
| Australia | DCS Prisoner Health Program, Transport costs, Other | -Prisoner health costs incurred by health departments or other agencies. | Breakdown of services |
| United States [2015] | -Medical supplies (meds)  -Staff  -Primary medical care  -Mental Health  -Dental  -Substance abuse treatment  Hospitalization, Other |  |  |
| UK: England and Wales [2016-17] | -Physical healthcare  -Mental healthcare |  | Breakdown of services |
| UK: England and Wales [1996-97] | -Medical supplies (meds)  -Staff  -Visiting Psychiatrists  -Consultants  -Nursing care  -Dental  Other: Optical, Occupational health |  |  |

Australia: Health Expenditure in prisons was disaggregated from total operating costs in Australia’s annual report. It includes the costs of the ‘Department of Correctional Services prisoner health program, medical services, transports to appointments, and other’. A limitation of the data is that it does not represent total expenditure in jurisdictions where prisoner health service costs are incurred by other agencies, departments, and/or private facilities. Additionally, some jurisdictions are not able to fully disaggregate this category of costs from other operating costs.

Belgium: A report by the Belgium KCE focused explicitly on health expenditure in prisons. The budget was divided into three major categories of costs: ‘staff/human resource costs, health care organization/equipment costs, and health care delivery/treatment costs (including external consultation)’. An additional breakdown, particularly of delivery costs, was as follows: ‘primary care costs, pharmacy-related costs, secondary care costs, mental health care costs, dental health care costs, and other.’ The publishers noted data sources were difficult to access and likely underestimated costs associated with psychosocial services.

South Africa: South Africa lists health expenditures in the Care theme (see definition in previous theme), within the sub-category of *Health and Hygiene Services*. This approximation represents costs of ‘primary health care, nutrition, hygiene/vaccinations, and medical supplies’. Psychological services were excluded from this category, and combined with pastoral and social work service costs.

Romania: The Romanian National Penitentiary Administration released a certified budget to the Ministry of Justice for the 2016 financial year. Healthcare expenditure within the correctional system was designated to 3 categories: ‘Medical supplies/medicines, Sanitary materials, and Disinfectants’.

Ireland: The Irish Prison Service released a financial spending report for 2013. ‘Medical care’ was listed as a current expenditure. The report did not include a description of offered health services.

UK: Scotland: The Scottish Parliament published a 2016-2017 report on *Healthcare in prisons*. From its survey, the healthcare budget for the financial year 2016-17 was reported in total. Healthcare services in prison include the following: ‘Primary medical care, in-house staff [doc/nurses/pharm], mental health, addiction treatment, screening/vaccinations, medical supplies, outside specialist/consultant psychiatrists, physiotherapists, dentists, opticians’.

UK: England and Wales: The National Audit Office reported the 2016-17 NHS England budget on ‘mental and physical healthcare’ in prisons to be 400 million pounds. The NHS estimate included all health services for the adult prison estate, by compiling a list of contracts that “included an element of healthcare in prisons and then adding up the amount spent on each of them in 2016-17.”

United States: A report issued by the Pew Charitable Trust found the median spending on prison healthcare in FY 2015 to be $5720 across 49 states. Costs for individual states are included. A separate survey of 10 states’ spending on inmate medical care, by the Pew Charitable Trust, found that between 2007-11, ‘37% of spending was attributable to General Medical Care, 20% to hospitalization, 14% to mental health care, 14% to pharmaceuticals, 5% to substance abuse treatment, 4% to health care administration, 4% to dental care, and 1% to other’.

India: According to the Ministry of Home Affairs, 5.9% of spending on inmates went to medical care in 2015-16. Expenses by *some* state prisons included the following services: ‘routine health check-ups, in-house treatment, screening for newly admitted inmates, doctors/nurses/pharmacists, and visiting psychologists to diagnose and treat mental disorders’.

Sri Lanka: The Sri Lankan Central Government manages financial schemes to cover hospitals under the Central Ministry of Health, Ministry of Defense, and Ministry of Justice [prison hospitals]. In the 2013 National Health Accounts of Sri Lanka, spending on healthcare is broken down by healthcare provider. Our estimate is based on the ‘prison hospital’ provider expense, which includes ‘specialized in-patient curative care, and in-patient curative and rehabilitative care’. Prison hospitals exclude costs of lab/imaging services, ancillary services, and certain medical supplies (i.e. glasses/hearing aids)
